# CWHM-1008 Induces Apoptosis and Protective Autophagy through the Akt/mTOR Axis in LUAD Cells

**DOI:** 10.1155/2021/5548128

**Published:** 2021-09-11

**Authors:** Jiao Zhang, Man-Yuan Li, Xiao Lu, Quan-Xing Liu, Dong Zhou, Gui-Xue Yang, Xiao-Qing Liu, Hong Zheng, Ji-Gang Dai

**Affiliations:** Department of Thoracic Surgery, Xinqiao Hospital, Army Medical University, Chongqing 400037, China

## Abstract

Recent studies have revealed that antiparasitic agents showed promising inhibitory effects on tumors, raising a possibility that repositioning this class of drugs may shed new light on clinical therapy against tumors. CWHM-1008 is a novel class of antimalarial drug; however, the inhibitory impact of CWHM-1008 on lung adenocarcinoma (LUAD) cells remains unclear. This study aimed to explore the anticancer effect and underlying mechanisms of CWHM-1008 on LUAD cells in vitro and in vivo. Human LUAD cells, H358 and A549, were treated with varying concentrations of CWHM-1008 at different lengths of time. Cell viability, colony formation, cell count, flow cytometry findings, microtubule-associated protein-1 light chain 3-green- (LC3-) GFP/RFP adenovirus infection status, and the expression of apoptosis and autophagy-related proteins were examined. Potential effects of an autophagy inhibitor (LY294002) and constitutively active Akt plasmid (CA-Akt) on CWHM-1008-induced apoptosis were also examined. Our results showed that CWHM-1008 significantly inhibited proliferation, induced apoptosis, and enhanced autophagy flux by blocking the RAC-alpha serine/threonine-protein kinase/the mammalian target of rapamycin (Akt/mTOR) axis in two LUAD cells. In addition, autophagy inhibited by LY294002 or CA-Akt transfection accelerated CWHM-1008-induced apoptosis in those LUAD cells. Moreover, CWHM-1008 significantly inhibited the growth and induced apoptosis of A549 cell in nude mice in vivo. The present findings provide new insights into anticancer properties of CWHM-1008, suggesting that it may be an adjuvant treatment for LUAD treatment, warranting further study.

## 1. Introduction

Lung cancer is associated with the highest incidence (11.6%) and mortality (18.4%) rates of all cancer types among both men and women worldwide; approximately 2.1 million patients develop this condition, and 1.8 million die from it annually [1]. Lung adenocarcinoma (LUAD) is the most common type of lung cancer, accounting for approximately 40% of all lung cancer cases [2] and creating a significant burden on healthcare systems. Although surgery, chemotherapy, targeted therapy, and immunotherapy, among other strategies, have been proposed to improve survival outcomes of LUAD [3–5], drug resistance, recurrence, and metastasis are associated with poor prognosis and high rates of treatment failure [6], resulting in 5-year survival rates of <15% [7]. Therefore, novel therapeutic agents against lung cancer are required.

Recently, drug repositioning has become an important strategy in the discovery and development of new anticancer drugs due to the low associated risk and cost. Approximately 235 nononcology agents have been associated with antitumor properties [8]. Antiparasitic drugs are safe and effective agents active against a wide range of internal and external parasites, widely used in both veterinary and medical practice. Among them, artemisinin, albendazole, and ivermectin have been shown to have strong anticancer properties in several cancer types [9–12]. CWHM-1008 is a pyrrolidine carboxamide chemotype agent that is potent and orally active with low molecular weight, mild lipophilicity, and a long half-life in mice, as well as remarkable antimalarial efficacy in vitro and in vivo [13]. In fact, previous studies have suggested that CWHM-1008 might be a suitable candidate for further development as a treatment agent for various diseases.

Autophagy and apoptosis are two pathways to cell death. Apoptosis plays a major role in the pathogenesis of diverse malignancies; it is a target of many antitumor agents [14]. Autophagy has recently emerged as a focus in cancer research; it plays a role in chemotherapy and has been shown to either suppress or promote the therapeutic effects of cancer treatments [15–17]. Meanwhile, the relationship between autophagy and apoptosis remains unclear. On the one hand, autophagy may suppress apoptotic cell death [18]; on the other hand, autophagy and apoptosis may cooccur, resulting in cell death [19], suggesting that the interplay between the two mechanisms warrants further research as a potential therapeutic strategy for cancer treatment. Autophagy is regulated by many signal transduction pathways [20]. Among these pathways, the RAC-alpha serine/threonine-protein kinase/the mammalian target of rapamycin (Akt/mTOR) pathway is one of the most vital regulators of autophagy, which also plays an important role in tumorigenesis and development [21, 22]. Inhibition of this pathway has been extensively shown to produce anticancer effects by inducing autophagy and apoptosis [23, 24]. Apatinib inhibited tumor growth by stimulating protective autophagy and apoptosis by blocking the Akt/mTOR pathway in anaplastic thyroid cancer [25]. Tanshinone I demonstrated its anticancer activity against ovarian cancer by inducing apoptosis and autophagy via the inactivation of the PI3K/Akt/mTOR pathway [26]. These studies demonstrated that autophagy regulation is a promising strategy to sensitize chemical and targeted drugs in tumor therapy [27].

Whether CWHM-1008, a potential antimalaria drug, could play an inhibitor role against LUAD cells is still unclear. In this study, we aimed to elucidate the anticancer properties and potential molecular mechanisms of CWHM-1008 in human LUAD cells, which could provide a foundation for further research into the therapeutic potential of CWHM-1008 for lung cancer.

## 2. Methods

### 2.1. Chemicals and Reagents

**Figure 1:**
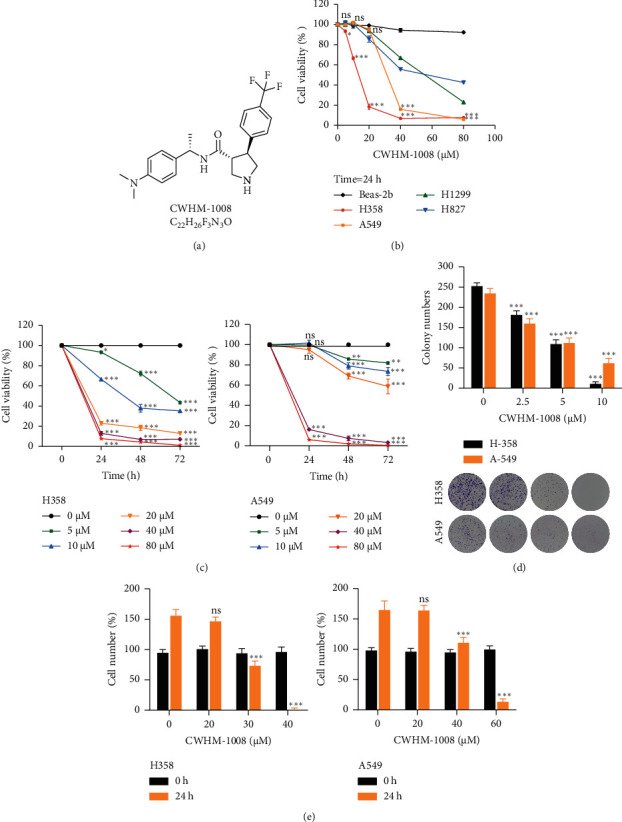
CWHM-1008 inhibits cell proliferation in human lung adenocarcinoma (LUAD) cells. (a) Chemical structure of CWHM-1008. (b) CWHM-1008 inhibits LUAD cell viability, evaluated using the Cell Counting Kit-8 assay in H358, A549, H-1299, H-827, and Beas-2b cells, treated with different concentrations of CWHM-1008 (0, 5, 10, 20, 40, or 80 *µ*M) for 24 h Data was compared with control (0 *µ*M) in each group. (c) The cell viabilities of H358 (up) and A549 (bottom) cells treated with different concentrations of CWHM-1008 for 0, 24, 48, and 72 h were evaluated using the Cell Counting Kit-8 assay. Data was compared with control (0 h in each group. (d) H358 and A549 cells were cultured at different concentrations of CWHM-1008 (0, 2.5, 5, or 10 *µ*M) for 10 days. Then, colony formation for each group was observed with crystal violet staining. Data was compared with control (treated with 0 *µ*M for 10 days) in each group. (e) H358 (left) and A549 (right) cells were cultured at various concentrations of CWHM-1008 (0, 20, 30, or 40 *µ*M) for 0 and 24 h. Cell number was first normalized to control (data at 0 h) in each group, and statistical analysis was performed between groups treated with various concentrations of CWHM-1008 and blank group (treated with 0 *µ*M for 24 h). Data are presented as the mean ± SD, *n* = 3. ^*∗*^*P* < 0.05, ^∗∗^*P* < 0.01, and ^∗∗∗^*P* < 0.001; ns, not significant.

CWHM-1008 (HY-111746) and LY-294002 (HY-10108) were purchased from MedChem Express Company (New Jersey, USA). The molecular structure of CWHM-1008 is shown in Figure 1(a). Dulbecco's modified eagle medium (DMEM) and Roswell Park Memorial Institute 1640 (RPMI 1640) medium, fetal bovine serum (FBS), and trypsin were purchased from HyClone (Buckinghamshire, UK). The bicinchoninic acid (BCA) protein assay kit, Cell Counting Kit-8 (CCK-8), terminal deoxynucleotidyl transferase-mediated nick-end labeling (TUNEL), dimethyl sulfoxide, penicillin-streptomycin, propidium iodide (PI), and APC-Annexin V were obtained from Beyotime (Shanghai, China). The rabbit anti-human Ki-67 monoclonal antibody was purchased from abcarta (Suzhou, China). Primary antibodies, including caspase-3 (9662S), cleaved-caspase-3 (C-caspase-3, 9661S), poly-ADP-ribose polymerase (PARP, 9532S), cleaved-PARP (C-PARP, 5625S), microtubule-associated protein-1 light chain-3 (LC3, 11972S), recombinant autophagy-related protein-7 (ATG7, 8558S), beclin-1 (3495S), sequestosome-1 (P62, 16177S), the RAC-alpha serine/threonine-protein kinase (Akt) (4685S), phospho-Akt (p-Akt) (4060S), mTOR (2983S), phosphorylated mTOR (p-mTOR) (5536S), and *β*-actin (4970S) were purchased from Cell Signaling Technology (Danvers Massachusetts, USA). Secondary antibodies (anti-rabbit, A0545) were purchased from Sigma Aldrich (Darmstadt, Germany).

### 2.2. Cell Culture

Human LUAD cell lines H358, A549, H1299, and H827 were obtained from the Cell Bank of the Chinese Academy of Sciences (Shanghai, China). Human normal lung epithelial cell line Beas-2b was purchased from Fengh Bio (Changsha, China). All cells were grown in DMEM or RPMI 1640, supplemented with 10% FBS (v/v) and 1% penicillin-streptomycin (v/v) at 37°C in a humidified atmosphere under 5% CO2.

### 2.3. Construction of Constitutively Active Akt (CA-Akt) Plasmid and Transfection

Coding sequence of human AKT1 was amplified from cDNA derived from A549 cells and inserted into pcDNA3.1. Point mutant plasmids pcDNA3.1-CA-Akt (T308D/S473D) were constructed using polymerase chain reaction. CA-Akt plasmids were extracted with Endo-Free Plasmid Midi Kit-fast (OMEGA, GA, USA) and used for transfection. 3 *μ*g CA-Akt or pcDNA3.1 vector was used for transfected H358 and A549 in a well of 6-well plate (1.5 *μ*g for a well of 12-well plate) by using lipofectamine 3000 reagent (Thermo Fisher Scientific, MA, USA).

### 2.4. Cell Viability Assay

Cell viability was assessed using the cerulein and cholecystokinin octapeptide (CCK-8) assay, whereby 5 × 10^3^ H358, A549, H-1299, H-827, and Beas-2b cells were seeded into a 96-well plate, incubated for 24 h, and then cultured at the indicated concentrations (0, 5, 10, 20, 40, or 80 *µ*M) of CWHM-1008 for 24, 48, and 72 h. Next, the cells were incubated at 37°C for 2–4 h after adding CCK-8 solutions (10 *µ*L per well). Subsequently, optical density (OD) was measured by the Spectramax I3 plate reader at 450 nm. The formula for viability was as follows: viability (%) = OD of treatment group/OD of control group × 100%.

### 2.5. Colony Formation Assay

A total of 1 × 10^3^ H358 and A549 cells were seeded into a 6-well plate and incubated overnight to allow adherence. Further, the cells were cultured at the indicated concentrations (0, 2.5, 5, and 10 *µ*M) of CWHM-1008 for 10 days; the medium was changed every 3 days. Next, the cells in 6-well plates were fixed with 4% paraformaldehyde for 15 min and stained with 0.1% crystal violet for 30 min before gently washing several times in phosphate buffer saline (PBS). Visible colonies over 50 cells were microscopically counted manually and photographed.

### 2.6. Cell Count Assay

A total of 5 × 10^5^ H358 and A549 cells were seeded into a 12-well plate and concurrently incubated with different concentrations of CWHM-1008 (0, 20, 30, or 40 *µ*M) for 0 and 24 h. The cells were then counted using a hemocytometer.

### 2.7. Flow Cytometry

A total of 5 × 10^5^ H358, A549, and Beas-2b cells were seeded into a 12-well plate and incubated overnight to allow adherence. Then, the cells were cultured at the different concentrations (0, 20, 30, or 40 *µ*M) of CWHM-1008 for 24 h. The cells were collected and washed several times with PBS and then resuspended with the Annexin V and PI for apoptosis analysis. Approximately 20,000 cells were examined by flow cytometry. The data were analyzed using FlowJo V10 software.

### 2.8. LC3-GFP/RFP Adenovirus Infection

1 × 10^5^ H358 and A549 cells per well were seeded into a 24-well plate and incubated for 24 h. The cells were then pretreated with 8 ug/mL polybrene and infected with LC3-GFP/RFP adenovirus. After 24 h, the H358 and A549 cells were incubated at the different concentrations of CWHM-1008 for 24 h. Next, the cells were fixed in 4% paraformaldehyde, washed several times with PBS, and then counterstained with 4′,6-diamidino-2-phenylindole (Beyotime Biotechnology, Shanghai, China) for 5 min and washed twice with PBS. An LSM 780 Meta confocal microscope (Carl Zeiss MicroImaging GmbH, Jena, Germany) was used to observe autophagosome accumulation.

### 2.9. Western Blotting

Twenty-four hours after the treatments with varying concentrations of CWHM-1008 alone or cotreatment with CWHM-1008 and 10 *µ*M LY294002, H358, and A549 cells were collected after trypsin digestion and washed with sterile PBS. The collected cells were lysed in lysis buffer plus protease inhibitor cocktail to obtain total protein. After incubation on ice for 15 min, the extracts were centrifuged at 12,000 rpm for 10 min at 4°C, and the supernatants were quantified using the BCA protein assay kit. Equal amounts of proteins (15 *µ*g/well) were separated by 10% sodium dodecyl sulfate-polyacrylamide gel electrophoresis and then transferred to the polyvinylidene fluoride membrane. The membranes were blocked with bovine serum albumin for 1 h at room temperature, followed by overnight incubation in the primary antibody at 4°C. After washing with phosphate buffered saline with 0.1% Tween 20 detergent, the membranes were incubated with secondary antibodies for 2 h at room temperature. The protein bands were detected by the chemiluminescent horseradish peroxidase substrate and compared with the reference protein (*β*-actin).

### 2.10. In Vivo Experiment

Female BALB/c and nude mice at 4 weeks of age were purchased from Vital River (Beijing, China) and acclimated for one week. All animal studies were approved by the Ethical Committee for Animal Experimentation of the Third Military Medical University. For acute oral toxic study, six female BALB/c mice were randomly divided into two groups (control and 300 mg/kg CWHM-1008) with 3 mice in each. 300 mg/kg CWHM-1008 prepared in olive oil was orally administered by gastric intubation on the first day; the control group was treated with the same volume of olive oil. Body weight and food intakes were measured daily for 14 days, and then weekly changes were recorded. Meanwhile, mortality (if any) and abnormity of mice were also recorded. All mice were sacrificed by decapitation at the end of administration, and the organs (liver, lung, heart, spleen, and kidney) were collected and weighed to calculate organ coefficients. In vivo anti-tumor efficacy was evaluated using subcutaneous A549 tumor-bearing nude mice. 5 × 10^6^ A549 cells were resuspended in 125 *µ*L PBS with 50% Matrigel (BD Corning, CA, USA) and injected subcutaneously into nude mice. When the tumor volumes reached 100 mm^3^, mice were randomized into three groups (4 mice per group) and orally dosed by gastric intubation receiving 0.1 mL olive oil, CWHM-1008 (15 mg/kg or 30 mg/kg) once every two days for 20 days. The tumor volume was measured with a caliper every 2 days using the formula: volume = length × width^2^. All mice were sacrificed by decapitation 20 days later; the tumor tissues were isolated, weighed and photographed, and fixed in 4% formalin.

### 2.11. Immunohistochemistry

Immunohistochemistry analysis of Ki-67 and TUNEL were performed as described previously [10]. The immunostaining intensity (*A*) was indicated by four grades (0, negative; 1, weakly positive; 2, positive; 3, strongly positive), and the proportion of staining-positive cells (*B*) was divided into five grades (0, <5%; 1, 6%–25%; 2, 26%–50%; 3, 51%–75%; 4, >75%). The final score was calculated as *A* × *B*. Images were captured using a DM2500 fluorescence microscope (Leica).

### 2.12. Statistical Analysis

All data are presented as mean ± standard deviation of findings from at least three repeat experiments; analyses were performed with SPSS 23.0 software. Data were assessed using a one-way analysis of variance and the Tukey or Games–Howell tests were used for comparisons. *P* values of <0.05 were considered indicative of a statistically significant finding.

## 3. Results

### 3.1. CWHM-1008 Inhibits Cell Proliferation and Induces Apoptosis in LUAD Cells

The chemical structure of CWHM-1008 is shown in Figure 1(a). To evaluate the anticancer effects of CWHM-1008 in LUAD cells, CCK-8 analysis was conducted to detect cell viability of four LUAD cell lines and a human normal lung epithelial cell line (Beas-2b) after treated with CWHM-1008. As shown in Figure 1(b), the cell viabilities of H358, A549, H1299, and H827 cells were inhibited to varying degrees in response to different concentrations of CWHM-1008 for 24 h; however, no significant change of cell viability in Beas-2b cell was observed. Moreover, cell viabilities of H358 and A549 cells were also reduced after CWHM-1008 treated in a time-dependent manner (Figure 1(c)). The antiproliferation activity of CWHM-1008 in H358 and A549 cells was further demonstrated by the reduced rate of clonogenicity survival (Figure 1(d)). In addition, CWHM-1008 treatment for 24 h significantly decreased the number of H358 and A549 cells as compared with that of control cells (Figure 1(e)).

**Figure 2:**
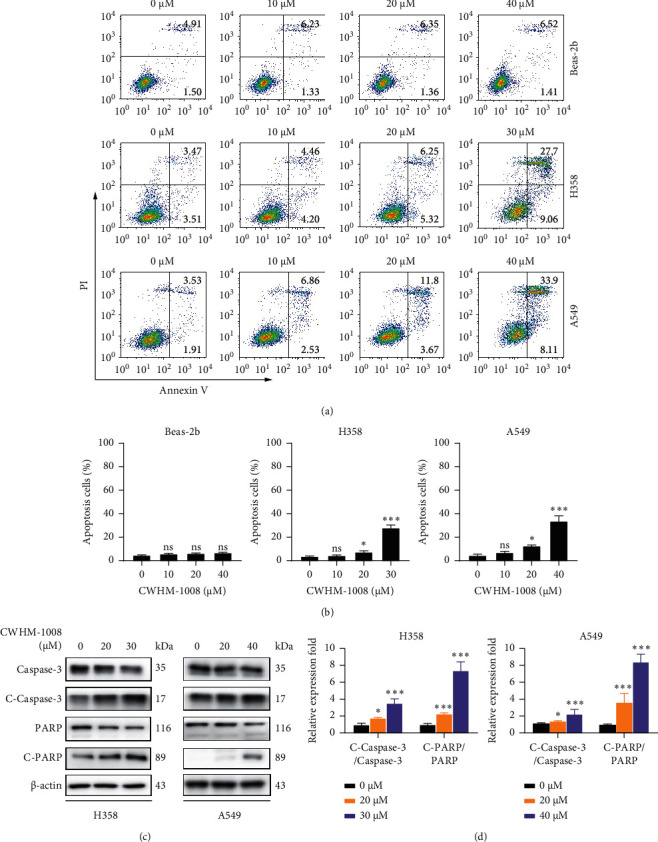
CWHM-1008 induces apoptosis in lung adenocarcinoma cells. (a) H358, A549, and Beas-2b cells were cultured at various concentrations of CWHM-1008 (0, 10, 20, 30, or 40 *µ*M) for 24 h. Apoptotic rates of the harvested cells were measured using Annexin V/propidium iodide and detected by flow cytometry. (b) Histograms show the percentages of apoptotic cells. (c) Levels of caspase-3, cleaved-caspase-3, poly-ADP-ribose polymerase (PARP), and cleaved-PARP in H358 and A549 cells cultured at the indicated concentrations of CWHM-1008 for 24 h were detected by western blotting. *β*-Actin was used as the reference protein. (d) Densitometry quantification of band intensities in C was calculated as shown in the histogram using ImageJ software. Data are presented as the mean ± SD; *n* = 3; ^*∗*^*P* < 0.05 and ^∗∗∗^*P* < 0.001; ns, not significant.

To investigate whether CWHM-1008 stimulates apoptosis in LUAD cells, we first measured the apoptotic rate by flow cemetery. As expected, CWHM-1008 treatment for 24 h stimulated apoptosis in a dose-dependent manner in H358 and A549 cells but had no obvious effect on apoptosis in Beas-2b cells (Figures 2(a) and 2(b)). This result was further confirmed by western blot analysis, which showed that CWHM-1008 treatment decreased the levels of caspase-3 and PARP and increased those of both C-caspase-3 and C-PARP in a dose-dependent manner in H358 and A549 cells (Figures 2(c) and 2(d)).

### 3.2. CWHM-1008 Enhances Autophagy through Blocking the Akt/mTOR Pathway in LUAD Cells

**Figure 3:**
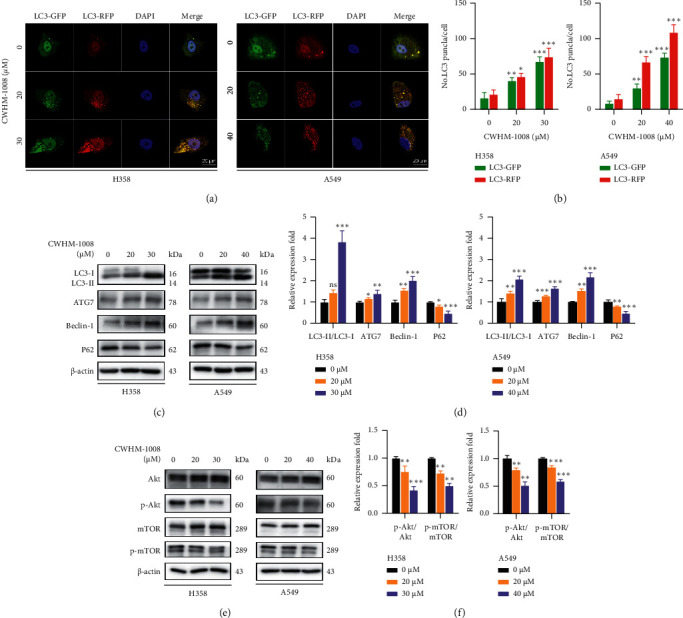
CWHM-1008 stimulates autophagy by blocking the Akt/mTOR axis in lung adenocarcinoma cells. (a) H358 (left) and A549 (right) cells were transiently infected with GFP-RFP-LC3 adenovirus. In addition, H358 and A549 cells were treated with various concentrations of CWHM-1008 (0, 20, 30, or 40 *µ*M) for 24 h. Scale bars, 20 *µ*m. (b) Total number of LC3-GFP and LC3-RFP puncta per cell. (c, d) Levels of autophagy-related protein LC3, ATG7, beclin-1, and P62 in H358 and A549 cells treated with various concentrations of CWHM-1008 for 24 h were analyzed by western blotting. (e, f) Levels of RAC-alpha serine/threonine-protein kinase (Akt), phospho-Akt, mammalian target of rapamycin (mTOR), and phosphorylated-mTOR in H358 and A549 cells treated with the indicated concentrations of CWHM-1008 for 24 h were analyzed by western blotting. Data are presented as the mean ± SD, *n* = 3. ^*∗*^*P* < 0.05, ^∗∗^*P* < 0.01, and ^∗∗∗^*P* < 0.001; ns, not significant.

To ascertain whether CWHM-1008 may regulate autophagy in lung cancer, LC3-GFP/RFP adenovirus was used for H358 and A549 cell infection. Confocal images revealed that the number of both green and red autolysosome puncta increased and more yellow puncta were observed after CWHM-1008 treatment, as compared to the control group (Figures 3(a) and 3(b)). Moreover, CWHM-1008 treatment upregulated the protein levels of LC3-II, ATG7, and beclin-1, in a dose-dependent manner compared to control cells. However, the expression levels of P62, a well-known autophagic substrate, were downregulated in a dose-dependent manner after treatment with CWHM-1008 (Figures 3(c) and 3(d)). It has previously been reported that phosphorylated Akt/mTOR plays an important role as a negative modulator in autophagy activation [28]; therefore, we investigated whether the Akt/mTOR pathway is associated with CWHM-1008-induced autophagy. Western blot analysis revealed that CWHM-1008 treatment decreased p-Akt and p-mTOR expression relative to that observed under control conditions, indicating CWHM-1008 could induce autophagy via inhibition of Akt/mTOR pathway in H358 and A549 cells (Figures 3(e) and 3(f)).

### 3.3. Inhibition of Autophagy Accelerates CWHM-1008-Induced Apoptosis in LUAD Cells

**Figure 4:**
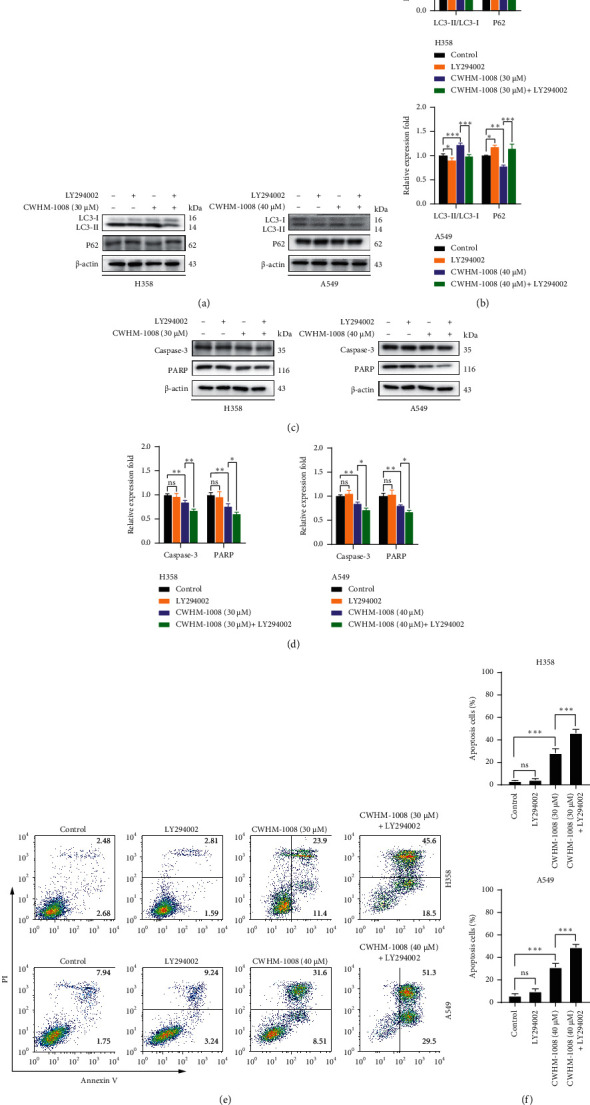
Suppression of autophagy accelerates the CWHM-1008-induced apoptosis in lung adenocarcinoma cells. (a–d) H358 and A549 cells were treated with the indicated concentrations of CWHM-1008 alone or pretreated with LY294002 (10 *µ*M) for 24 h and then the levels of autophagy-related protein LC3 and P62, and apoptosis-related protein caspase-3 and PARP were analyzed using western blotting. Densitometry quantification of the band intensities in A and C was calculated as shown in the histogram using ImageJ software. (e) Apoptotic rates of H358 (up) and A549 (bottom) cells treated with the indicated concentrations of CWHM-1008 alone or pretreated with LY294002 (10 *µ*M) for 24 h were detected by flow cytometry. (f) Histograms show the percentages of apoptotic cells. Data are presented as the mean ± SD, *n* = 3. ^*∗*^*P* < 0.05, ^∗∗^*P* < 0.01, and ^∗∗∗^*P* < 0.001; ns, not significant.

**Figure 5:**
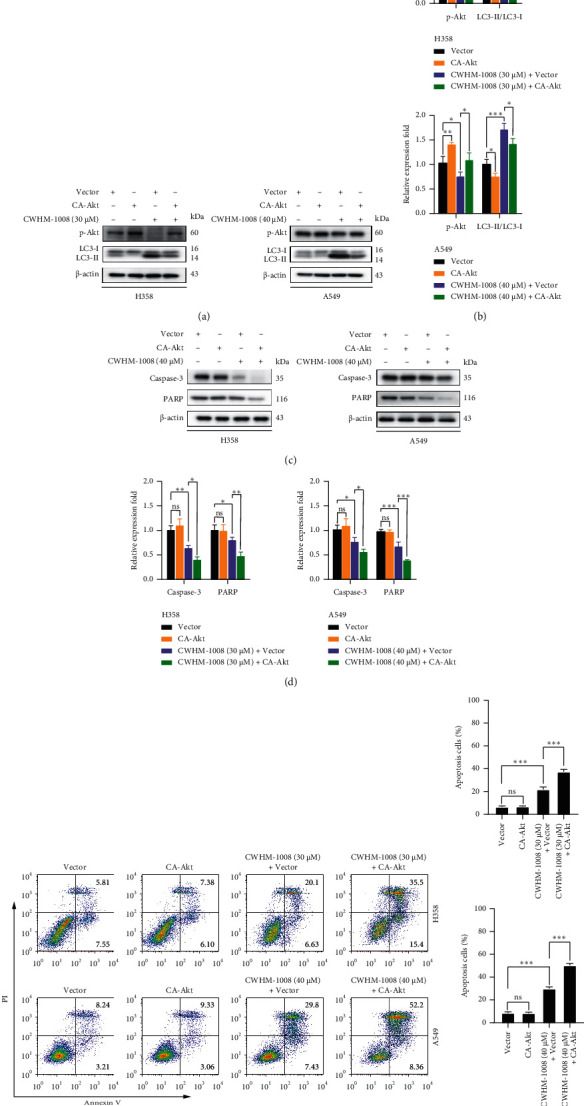
Constitutively active Akt facilitates the CWHM-1008-induced apoptosis in LUAD cells. (a–d) H358 and A549 cells were transfected with 3 *µ*g CA-Akt plasmids or control vector (pcDNA3.1) and then treated with the indicated concentrations of CWHM-1008 for 24 h, and then the levels of autophagy-related protein LC3 and p-Akt, and apoptosis-related protein caspase-3 and PARP were analyzed using western blotting. Densitometry quantification of the band intensities in A and C was calculated as shown in the histogram using ImageJ software. (e) H358 and A549 cells were transfected with 3 *µ*g CA-Akt plasmids or control vector and then treated with the indicated concentrations of CWHM-1008 for 24 h. Then, apoptotic rates of two cells were detected by flow cytometry. (f) Histograms show the percentages of apoptotic cells. Data are presented as the mean ± SD, *n* = 3. ^*∗*^*P* < 0.05, ^∗∗^*P* < 0.01, and ^∗∗∗^*P* < 0.001; ns, not significant.

To further study the possible link between apoptosis and autophagy induced by CWHM-1008, LY294002, an autophagy inhibitor [29], was used. Western blot analysis indicated that the treatment of CWHM-1008 with LY294002 significantly downregulated the LC3-II protein and upregulated P62, indicating that LY294002 treatment suppressed CWHM-1008-induced autophagy (Figures 4(a)4(b)). Meanwhile, LY294002 treatment significantly decreased the protein levels of caspase-3 and PARP (Figures 4(c) and 4(d)), and flow cytometry further confirmed the addition of LY294002 could substantially increase the rate of apoptosis in CWHM-1008-treated cells (Figures 4(e) and 4(f)). Moreover, CA-Akt plasmids were transfected into H358 and A549 LUAD cells to further validate the role of the Akt/mTOR pathway in CWHM-1008-induced apoptosis. As shown in Figure 5(a), p-Akt protein level significantly increased in LUAD cells after transfection with CA-Akt plasmids. CA-Akt transfection noticeably decreased LC3 protein and inhibited CWHM-1008-induced autophagy (Figures 5(a) and 5(b)), resulting in decreased caspase-3 and PARP protein (Figures 5(c) and 5(d)). Results of flow cytometry also confirmed that transfection of CA-Akt could facilitate the CWHM-1008-induced apoptosis in two LUAD cells (Figures 5(e) and 5(f)).

### 3.4. CWHM-1008 Inhibits Growth and Induces Apoptosis of A549 Cells In Vivo

**Figure 6:**
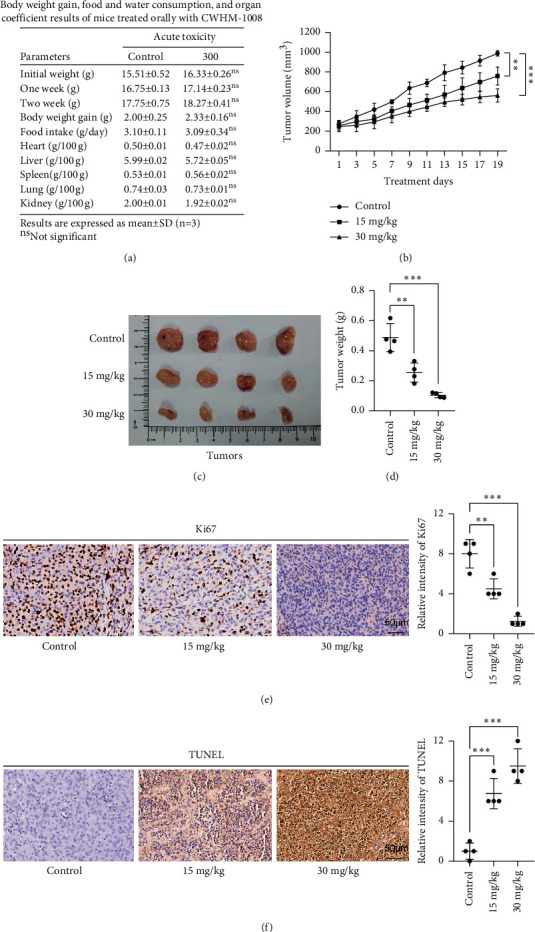
CWHM-1008 induced apoptosis of LUAD cell in vivo. (a) Body weight gain, food consumption, and organ coefficient results of mice treated orally with CWHM-1008. Oral administration of 300 mg/kg for one time and related data were collected 14 days after treatment. (b) Tumor volume curve of subcutaneous A549 tumor-bearing mice after receiving various dose of CWHM-1008 treatments. (c) Representative images for subcutaneous xenograft tumors; *n* = 4. (d) Tumor weights at time of sacrifice. Representative immunohistochemical pictures of Ki67 ((e), left) and TUNEL ((f), left) for each treatment group. Scale bar = 50 *μ*m. The scatter plot graph on the right shows the immunohistochemical score for Ki67 and TUNEL for each treatment groups. Data are presented as the mean ± SD, *n* = 4. ^∗∗^*P* < 0.01 and ^∗∗∗^*P* < 0.001; ns, not significant.

To further support our analysis data above, subcutaneous tumor experiments were performed. Acute toxicity study was first conducted to evaluate in vivo toxicity of CWHM-1008, and one-time oral administration of 300 mg/kg CWHM-1008 (10-fold higher than the conventional dose for malaria treatment in mice) did not show any toxicities to heart, lung, liver, spleen, and kidney and not cause death in any mouse, even in 14 days after treatment. Furthermore, CWHM-1008 did not affect food intake or body weight gain (Figure 6(a)). We then applied two doses of CWHM-1008 (15 and 30 mg/kg) throughout our subsequent in vivo experiments. As shown in Figures 6(b)−6(d), oral administration of CWHM-1008 every other day significantly inhibited the growth of A549 cells in nude mice in a dose-dependent manner. Immunohistochemistry for Ki67 and TUNEL revealed that CWHM-1008 treatment significantly decreased the proliferation index Ki67 (Figure 6(e)) and increased the apoptosis-related TUNEL index (Figure 6(f)).

## 4. Discussion

With the advancement of therapeutic strategies, epidermal growth factor receptor tyrosine kinase inhibitors (EGFR-TKI) and immune checkpoint inhibitors were used for the first-line treatment of patients with lung cancer [30, 31]. However, only some patients could benefit from these two treatments due to rapid drug resistance and low response rate of monotherapy [32, 33]. New adjuvant therapy agents for lung cancer are urgently required. In recent years, antiparasitic drugs have been shown promising anticancer properties. Ivermectin, a broad-spectrum antiparasitic drug, inhibited breast cancer cell growth by activating cytostatic autophagy by promoting ubiquitination degradation of PAK1 [10]. The antiparasitic agent suramin may kill cancer cells by blocking minichromosome maintenance protein 10-dependent DNA replication [34]. Therefore, these findings provide a new strategy to search for anticancer drugs among antiparasitic agents [12]. CWHM-1008 is a potent and orally active antimalarial drug with low molecular weight, mild lipophilicity, long half-life in mice, and notable antimalarial efficacy in vitro and in vivo [13]. The present study is the first to reveal remarkable anticancer properties of CWHM-1008 in lung cancer cells in vitro and in vivo. Results from CCK-8, flow cytometry, and xenograft experiments demonstrated that CWHM-1008 promoted apoptosis and growth inhibition of LUAD cells. The acute toxicity tests showed that at the tested doses, neither toxic symptoms nor death and no obvious behavioral changes were observed in any mice. Therefore, CWHM-1008 can be considered nontoxic at this effective dose. These findings indicate that CWHM-1008 might have prospects for further development.

Apoptosis induction in cancer cells is an anticancer mechanism shared by many agents [35]. Paclitaxel, a well-known anticancer drug, can induce apoptosis against tumor growth, but drug resistance limited its application in cancer treatment. Wu et al. reported that praziquantel, an antiparasitic agent, could markedly enhance the anticancer efficacy of paclitaxel in various cancer cells by activating the apoptotic cascade [36]. In our study, flow cytometry showed that CWHM-1008 may cause cellular apoptosis in H358 and A549 cells. Moreover, caspase and PARP family proteins are paramount to apoptosis [37], and western blot analysis showed that CWHM-1008 treatment significantly upregulated the levels of C-caspase-3 and C-PARP and downregulated those of caspase-3 and PARP. These data have shown that CWHM-1008 could induce caspase cascade reaction. Based on these results, this study indicated that the combination of CWHM-1008 and autophagy inhibitors may represent a novel and effective anticancer strategy.

Autophagy is a fundamental biological process that eliminates protein aggregates and damaged organelles, characterized by the formation of double-membrane autophagosomes [38]. Many studies have demonstrated that autophagy is involved in the progression of cancers. Four different forms of autophagy are involved in the improvement of cancer therapy, namely, cytoprotective, cytostatic, cytotoxic, and nonprotective autophagy [39]. However, the mechanism of autophagy regulation in drug-resistant tumor cells remains unclear. On the one hand, inhibiting autophagy can enhance sensitivity to chemotherapy and promote apoptosis in cancer cells; on the other hand, inducing autophagy can also inhibit the growth of tumors [17, 40]. This evidence suggests the necessity to study multiple mechanisms of autophagy before they can be used as therapeutic targets in cancer care. To our knowledge, the present study is the first to report that CWHM-1008 may induce autophagy in human LUAD cell lines, using LC3-GFP/RFP adenovirus status and western blotting analysis of the key autophagy-related proteins. Despite their different impact on the survival of cancer cells, the functions of autophagy and apoptosis are not completely independent. Consequently, to clarify the correlation, CWHM-1008 was used in combination with the autophagy inhibitor LY294002. Our results showed that LY294002-mediated autophagy may suppress the rate of CWHM-1008-induced apoptosis, suggesting that CWHM-1008-induced autophagy plays a protective role against apoptosis in LUAD cells. Interestingly, chloroquine, as an FDA-approved antimalarial chemical and autophagy inhibitor, is currently the principal compound used in clinical trials aimed to treat tumors [41]. Different regulatory roles in autophagy of these two antimalaria drugs suggested that the combination treatment of CWHM-1008 and chloroquine may be a new potential therapeutic strategy for the treatment of lung cancer.

The Akt/mTOR pathway is an important negative pathway in the activation of autophagy; it is also involved in regulating cancer cell proliferation, migration, cell cycle, and apoptosis [22, 24, 42–44]. Previous studies have investigated antiparasitic agents, including itraconazole and ivermectin, which block the Akt/mTOR pathway, thereby inducing autophagy and leading to cancer cell death [10, 45]. However, the present findings suggest that CWHM-1008 could decrease the levels of p-Akt and p-mTOR, and decreased phosphorylation of Akt and mTOR can induce autophagy [46]. Different from itraconazole and ivermectin, we found that the Akt/mTOR signaling pathway activated by CA-Akt transfection accelerated CWHM-1008-induced apoptosis in two LUAD cells, revealing that CWHM-1008 could mediate protective autophagy against cell death. Moreover, the underlying mechanisms for different functions of autophagy induced by these antiparasitical agents and CWHM-1008 mediated p-AKT inhibition warrant further investigation.

In summary, the present findings suggest that CWHM-1008 may induce apoptosis and protective autophagy by acting on the Akt/mTOR signaling pathway in LUAD cells. The present study provides novel insights into the anticancer properties of CWHM-1008 and proposes a theoretical foundation for the clinical evaluation of CWHM-1008 combined with autophagy inhibitors as an adjuvant therapeutic strategy for lung cancer.

## Data Availability

All data that support the findings of the current study are included within the article.
